# Low Ankle-Brachial Index in Patients on Hemodialysis Is Associated with Elevated Serum Levels of Spermine Oxidase and C-Reactive Protein

**DOI:** 10.3390/life16091521

**Published:** 2026-09-13

**Authors:** Yahn-Bor Chern, Jen-Pi Tsai, Yu-Chi Chang, Po-Yu Huang, Hung-Hsiang Liou, Bang-Gee Hsu

**Affiliations:** 1 Division of Nephrology, Department of Medicine, Yuan’s General Hospital, Kaohsiung 802635, Taiwan; 2 Division of Nephrology, Department of Internal Medicine, Dalin Tzu Chi Hospital, Buddhist Tzu Chi Medical Foundation, Chiayi 62247, Taiwan; 3 School of Medicine, Tzu Chi University, Hualien 97004, Taiwan; 4 Institute of Medical Sciences, Tzu Chi University, Hualien 97004, Taiwan; 5 Division of Nephrology, Hualien Tzu Chi Hospital, Buddhist Tzu Chi Medical Foundation, Hualien 97004, Taiwan; 6 Division of Nephrology, Department of Internal Medicine, Hsin-Jen Hospital, New Taipei City 24243, Taiwan

**Keywords:** spermine oxidase, peripheral arterial disease, hemodialysis, ankle–brachial index, C-reactive protein

## Abstract

Peripheral arterial disease (PAD) is a major cardiovascular complication of hemodialysis (HD), during which inflammation and oxidative stress contribute to vascular injury. The role of spermine oxidase (SMOX), a polyamine-metabolizing enzyme generating hydrogen peroxide, in PAD is unknown in HD. This cross-sectional study included 142 patients on maintenance HD, including 115 with a normal ankle–brachial index (ABI) (≥0.9) and 27 with a low ABI (<0.9). Patients with a low ABI were older and had lower creatinine and albumin levels, fractional clearance index for urea, and urea reduction ratio but higher levels of glucose, C-reactive protein (CRP), and SMOX; diabetes mellitus was also more prevalent (*p* < 0.05 for all). In Firth-penalized multivariable logistic regression, SMOX (adjusted odds ratio [aOR] per 1 ng/mL increase, 1.063; 95% confidence interval [CI], 1.018–1.115; *p* = 0.001) and CRP (aOR per 0.1 mg/dL increase, 1.181; 95% CI, 1.079–1.321; *p* < 0.001) were independently associated with ABI-defined PAD. Conventional and penalized sensitivity analyses yielded similar estimates. The areas under the curve for SMOX and CRP were 0.790 and 0.804, respectively. SMOX was inversely correlated with bilateral ABI and positively correlated with CRP. SMOX may represent a candidate biomarker associated with ABI-defined PAD in patients undergoing HD, although further validation in larger prospective cohorts is required.

## 1. Introduction

Despite advances in therapeutic interventions, cardiovascular disease (CVD) remains a major cause of morbidity and mortality among patients with end-stage renal disease on maintenance hemodialysis (HD) [1], who also experience peripheral arterial disease (PAD), a major cardiovascular complication [2]. In patients on HD, the PAD presentation extends well beyond local limb ischemia and is a strong indication of systemic atherosclerosis and an important contributing factor to total cardiovascular burden [3]. Individuals on HD experience an accelerated clinical course of PAD, associated with debilitating consequences of chronic pain, poor wound healing, high rates of lower extremity amputation, and increased cardiovascular mortality [4,5].

PAD is common in patients with chronic kidney disease (CKD) and end-stage renal disease. This burden is related to a combination of classic cardiovascular risk factors and CKD-specific alterations related to uremia, in which sustained systemic inflammation and severe oxidative stress are key players [6,7]. Collectively, these processes aggravate endothelial dysfunction, accelerate atherosclerosis progression, and induce vascular calcification [8,9]. Consequently, vascular homeostasis is compromised, leading to progressive arterial stiffening and luminal narrowing. Thus, understanding the metabolic pathways underlying PAD can provide key insights into accelerated vascular aging commonly observed in patients on HD.

Existing circulating and clinical markers, including C-reactive protein (CRP), galectin-3, and uremic toxin-related markers, have been investigated in relation to PAD or vascular dysfunction in patients with CKD or HD [10,11,12,13]. However, these markers mainly reflect systemic inflammation, endothelial injury, or uremic burden, and may not fully capture oxidative stress-related metabolic signals associated with vascular impairment in HD. Therefore, evaluating serum spermine oxidase (SMOX) may provide additional insight into a less characterized oxidative stress-related biomarker associated with ABI-defined PAD in this population, as dysregulated polyamine metabolism can contribute to oxidative stress through SMOX-mediated generation of hydrogen peroxide and reactive aldehydes.

One potential pathway that may be relevant to PAD pathogenesis is polyamine metabolism, which is regulated by SMOX and other enzymes. At physiological conditions, SMOX catalyzes the conversion of spermine to spermidine, a cellular pathway critical for proper growth, differentiation, and cellular metabolism [14]. In contrast, in pathological states, SMOX overactivity may increase the production of toxic metabolites, particularly reactive aldehydes and hydrogen peroxide [15], and the overproduction of reactive oxygen species can exceed the antioxidant capacity threshold, potentially contributing to oxidative stress and cellular damage [16,17]. SMOX-mediated oxidative stress has been implicated in vascular endothelial injury, inflammation, and atherogenesis [18,19]. However, its mechanistic role in PAD among patients undergoing HD remains uncertain, and its association with PAD in this population has not been established.

Given the ongoing need to identify biomarkers for vascular risk in patients on HD, the present study aimed to evaluate the relationship between SMOX and PAD by assessing the association of PAD, defined using the ankle–brachial index (ABI), with serum SMOX and CRP levels.

## 2. Materials and Methods

### 2.1. Study Design and Patient Selection

The present cross-sectional study received ethical approval from the Research Ethics Committee of Hualien Tzu Chi Hospital, Buddhist Tzu Chi Medical Foundation (approval no: IRB108-219-A), and written informed consent was obtained from all participants. Using purposive sampling, participants were recruited from the outpatient HD unit of a medical center in Hualien, Taiwan, between 1 May 2022, and 31 August 2022. Eligible patients were aged ≥20 years who were on maintenance HD three times/week with standard bicarbonate dialysate for at least 3 months. All participants underwent HD using a high-flux polysulfone disposable artificial kidney (FX-class dialyzer; Fresenius Medical Care, Bad Homburg, Germany). Patients with an active infection, malignancy, a history of amputation or stroke, cirrhosis, chronic obstructive pulmonary disease, chronic heart failure, or pulmonary edema at the time of blood sampling, those who were bedridden at the time of blood sampling, those with an ABI > 1.3, and those who were unable to give consent were excluded from the study. Information regarding previous SARS-CoV-2 infection, asymptomatic infection, and COVID-19 vaccination status was not systematically collected. Four patients undergoing HD were excluded because their ABI was >1.3 in at least one lower extremity. Among these four patients, two were male and two were female. Their mean age was 43.00 ± 10.89 years, and the median HD duration was 206.04 months (range, 47.40–292.80 months). None had diabetes mellitus, and one patient (25.0%) had hypertension. After application of the study exclusion criteria, 142 patients undergoing HD were included in the final analysis.

### 2.2. Clinical Measurements and Definitions

Blood pressure (BP) was measured in the morning by the same trained personnel using a mercury sphygmomanometer with properly sized cuffs, after the patient had rested for at least 30 min. Systolic and diastolic BP were measured at the onset and cessation of Korotkoff sounds, respectively. The average of three measurements taken at 5-min intervals was used for analysis. Hypertension was defined as a systolic BP ≥ 140 mmHg, diastolic BP ≥ 90 mmHg, or current use of antihypertensive medications, according to the Eighth Joint National Committee guidelines. Diabetes mellitus (DM) was defined as a fasting plasma glucose level ≥ 126 mg/dL, a hemoglobin A1c ≥ 6.5%, or current use of antidiabetic agents.

### 2.3. Anthropometric Analysis

All anthropometric measurements were performed by the same trained operator to reduce interobserver variability. In all patients, pre- and post-HD body weights were measured to the nearest 0.5 kg, with light clothing and no shoes. Height was measured to the nearest 0.5 cm. Body mass index was calculated as post-HD body weight divided by height squared [10,20].

### 2.4. Biochemical Measurements

Approximately 5 mL of venous blood was collected from all patients before the midweek HD session (Tuesday or Wednesday) for primary biochemical profiling. Approximately 0.5 mL of the collected blood sample was used for a complete blood cell count determined with a Sysmex XS-1000i analyzer (Sysmex America, Mundelein, IL, USA). The leftover blood sample was immediately centrifuged at 3000× *g* for 10 min. The separated serum was maintained at 4 °C and analyzed within 1 h for routine biochemical testing, whereas additional serum aliquots were stored at −80 °C until SMOX measurement. Serum concentrations of blood urea nitrogen (BUN), creatinine, albumin, glucose, total cholesterol (TCH), triglycerides, total calcium, phosphorus, and CRP were measured using an autoanalyzer (Siemens Advia 1800; Siemens Healthcare GmbH, Erlangen, Germany). Serum levels of intact parathyroid hormone (iPTH) and SMOX were measured with commercially available enzyme-linked immunosorbent assay kits (catalog no: ab230931; Abcam, Cambridge, MA, USA; and catalog no: MBS9714233; MyBioSource, San Diego, CA, USA, respectively) [10,20]. According to the manufacturer’s documentation for the SMOX assay kit used in this study, the analytical measurement range was 0.156–10 ng/mL, and the lower limit of detection was 0.059 ng/mL. The lower limit of quantification was not specified by the manufacturer. Serum samples were diluted 20-fold by mixing one volume of serum with 19 volumes of the supplied sample diluent. Therefore, after correction for dilution, the reportable concentration range in the original serum samples was 3.12–200 ng/mL. Accordingly, the corresponding concentrations in the diluted samples ranged from 0.314 to 7.484 ng/mL, which were within the manufacturer-specified calibration range of 0.156–10 ng/mL. Thus, no reported SMOX value was calculated by extrapolation beyond the standard curve. Laboratory personnel performing the SMOX measurements were blinded to the participants’ ABI status to minimize measurement bias. Dialysis adequacy was assessed using the fractional clearance index for urea (Kt/V) and urea reduction ratio (URR), calculated from a formal single-compartment urea kinetic model based on pre- and post-dialysis BUN levels routinely obtained during dialysis sessions.

### 2.5. ABI Measurements

The ABI was assessed using an automated oscillometric device capable of simultaneous BP measurement across four extremities (VaSera VS-1000; Fukuda Denshi, Tokyo, Japan) [10,11], which was used to define PAD. Briefly, patients remained resting in the supine position for at least 30 min while wearing occlusion and monitoring cuffs with continuous electrocardiogram and heart sound recordings. The ABI for each lower extremity was calculated by dividing the ankle systolic BP by the arm systolic BP, using the lower value of the ankle systolic BP. Two measurements obtained from each lower extremity were used to calculate the mean ratio as the final ABI. Since an ABI of <0.9 is a widely accepted diagnostic criterion for PAD, patients with a low ABI were defined as those with a left or right ABI of <0.9 [21].

### 2.6. Statistical Analysis

First, normality of the data was assessed using the Kolmogorov–Smirnov test. Normally and non-normally distributed continuous variables were presented as means ± standard deviation and median (interquartile range), respectively, and compared using the two-tailed independent *t* and the Mann–Whitney *U* tests, respectively. Categorical data were presented as frequencies with percentages and compared using the χ^2^ test.

In the study cohort, only 27 participants had ABI-defined PAD; therefore, multivariable analysis was restricted to three prespecified predictors, namely, age, serum SMOX, and CRP, to reduce the risk of overfitting. These predictors were selected a priori based on the study hypothesis and biological plausibility rather than solely on univariable statistical significance. Serum SMOX was the main biomarker of interest, CRP was included as an established inflammatory marker and comparator related to vascular risk in CKD and HD, and age was included as a fundamental demographic risk factor for PAD and a potential confounder. Firth-penalized logistic regression was used as the primary analysis to reduce small-sample bias in maximum-likelihood estimates. As a sensitivity analysis, a conventional multivariable logistic regression model including the same three predictors was performed. Although other clinically relevant variables, including DM, glucose, albumin, creatinine, Kt/V, and URR, differed between ABI groups or correlated with ABI, adding these variables to the primary model would have resulted in an unfavorable events-per-variable ratio and unstable estimates. Therefore, these variables were described in the between-group and correlation analyses and further evaluated only in an exploratory expanded model. Additionally, LASSO (least absolute shrinkage and selection operator), ridge, and elastic-net logistic regression models were used as exploratory sensitivity analyses to assess the stability of associations under different coefficient shrinkage approaches. For this purpose, 5000 stratified bootstrap samples were used to estimate exploratory confidence intervals and *p*-values.

To further assess the robustness of the primary findings while limiting model complexity and overfitting, reduced Firth-penalized logistic regression sensitivity models were constructed. All reduced models included age, serum SMOX, and CRP. DM, albumin, and Kt/V were then added separately, one covariate at a time. URR was not included because of its strong mathematical and physiological overlap with Kt/V. Adjusted odds ratios (aORs) were reported for the specified increments, with profile-penalized likelihood 95% confidence intervals (CIs) and penalized likelihood ratio *p*-values. Variance inflation factors (VIFs) were calculated using auxiliary linear regression models containing the same predictors.

Spearman’s rank correlation coefficients were calculated to explore associations among ABI, serum SMOX, CRP, and clinical parameters. Binary logistic regression models incorporating serum SMOX and CRP levels were constructed to estimate the probability of PAD. Model discrimination was assessed using the area under the receiver operating characteristic curve (AUC). The optimal cutoff value for each biomarker was selected by maximizing the Youden index. Sensitivity, specificity, positive predictive value, and negative predictive value were calculated at the selected cutoff values. Model calibration was evaluated using the Brier score, Hosmer–Lemeshow goodness-of-fit test, calibration intercept, and calibration slope. For discrimination models of SMOX, CRP, and SMOX in combination with CRP, 5000 stratified bootstrap samples were used to estimate the optimism-corrected AUC, Brier score, calibration intercept, and calibration slope.

All statistical analyses were performed using Statistical Package for the Social Sciences for Windows version 25.0 (IBM, Armonk, NY, USA) and R version 4.2.2 (R Foundation for Statistical Computing, Vienna, Austria). A two-sided *p*-value of <0.05 was considered statistically significant.

## 3. Results

### 3.1. Descriptive Statistics and Between-Group Comparisons

The cohort included 77 men (54.2%) and 65 women (45.8%) on HD, including 27 (19.0%) patients with a low ABI (<0.9) and 115 (81.0%) patients with a normal ABI (≥0.9). Compared with the patients with normal ABI, those with a low ABI were significantly older (*p* = 0.002), had lower Kt/V (*p* < 0.001), URR (*p* < 0.001), serum creatinine (*p* = 0.008), and albumin (*p* = 0.031). In contrast, serum glucose, CRP, and SMOX levels were significantly higher in patients with a low ABI than in those with a normal ABI (*p* = 0.005, *p* < 0.001, and *p* < 0.001, respectively). The prevalence of DM was also higher among the patients with a low ABI (*p* = 0.015). Conversely, we did not observe significant differences in HD duration, sex, smoking, pre-HD body weight, body mass index, systolic and diastolic BP, hemoglobin, TCH, triglycerides, BUN, calcium, phosphorus, iPTH, hypertension, and glomerulonephritis between the two groups (Table 1). There were no significant differences between the normal ABI and low ABI groups in the use of ARBs, β-blockers, calcium channel blockers, statins, or fibrates.

### 3.2. Primary Multivariable Regression Analysis

In the primary Firth-penalized multivariable logistic regression model adjusted for age, higher serum SMOX and CRP levels were independently associated with PAD. Each 1 ng/mL increase in SMOX was associated with a 6.3% increase in the odds of PAD (aOR, 1.063; 95% CI, 1.018–1.115; *p* = 0.001), whereas each 0.1 mg/dL increase in CRP was associated with an 18.1% increase in the odds of PAD (aOR, 1.181; 95% CI, 1.079–1.321; *p* < 0.001). In contrast, age was not significantly associated with PAD (aOR per year, 1.041; 95% CI, 0.995–1.094; *p* = 0.082). In the conventional multivariable logistic regression model including age, serum SMOX, and CRP, higher SMOX levels were independently associated with PAD (aOR per 1 ng/mL increase, 1.067; 95% CI, 1.019–1.117; *p* = 0.006). Higher CRP levels were also independently associated with PAD (aOR per 0.1 mg/dL increase, 1.198; 95% CI, 1.079–1.330; *p* < 0.001). In contrast, age was not significantly associated with PAD (aOR per year, 1.044; 95% CI, 0.994–1.096; *p* = 0.083) (Table 2).

In the reduced Firth-penalized sensitivity models, serum SMOX and CRP remained significantly associated with ABI-defined PAD after the separate addition of diabetes mellitus, albumin, or Kt/V to the primary model containing age, SMOX, and CRP (Supplementary Table S1). The aORs for SMOX ranged from 1.063 to 1.071 per 1 ng/mL increase, with all *p* values ≤ 0.008, whereas those for CRP ranged from 1.178 to 1.199 per 0.1 mg/dL increase, with all *p* values < 0.001. Diabetes mellitus was associated with ABI-defined PAD in its respective model (aOR, 4.997; 95% CI, 1.575–19.625; *p* = 0.005), whereas albumin was not (aOR, 0.569; 95% CI, 0.203–1.489; *p* = 0.254). Higher Kt/V was inversely associated with ABI-defined PAD (aOR per 0.1-unit increase, 0.204; 95% CI, 0.062–0.505; *p* < 0.001). All predictor VIFs were ≤1.193, indicating no substantial multicollinearity in the reduced models.

### 3.3. Penalized Logistic Regression Sensitivity Analyses

The associations of SMOX and CRP with ABI-defined PAD remained consistent across the LASSO, ridge, and elastic-net models, whereas age remained nonsignificant in all penalized analyses (Table 3).

### 3.4. Correlation Analysis

Spearman’s rank correlation analysis to evaluate the associations of left-side ABI, right-side ABI, and serum SMOX level with clinical variables in 142 patients on HD revealed that left- and right-side ABIs were strongly and positively correlated with each other (ρ = 0.822, *p* < 0.001) (Table 4). In contrast, serum SMOX level was inversely correlated with both left-side ABI (ρ = −0.516, *p* < 0.001) and right-side ABI (ρ = −0.484, *p* < 0.001). Both left- and right-side ABI were negatively correlated with age, glucose, and CRP levels and positively correlated with serum creatinine and albumin levels (*p* < 0.05 for all). Right-side ABI was also positively correlated with diastolic BP (ρ = 0.191, *p* = 0.023). In contrast, serum SMOX level was positively correlated with age (ρ = 0.181, *p* = 0.031) and CRP (ρ = 0.308, *p* < 0.001) but negatively correlated with diastolic BP (ρ = −0.202, *p* = 0.016), creatinine (ρ = −0.255, *p* = 0.002), and albumin (ρ = −0.169, *p* = 0.044). No significant correlations were observed with body mass index, HD duration, systolic BP, hemoglobin, lipid profile, BUN, calcium, phosphorus, iPTH, Kt/V, or URR.

### 3.5. Model Discrimination, Calibration, and Internal Validation

**Figure 1:**
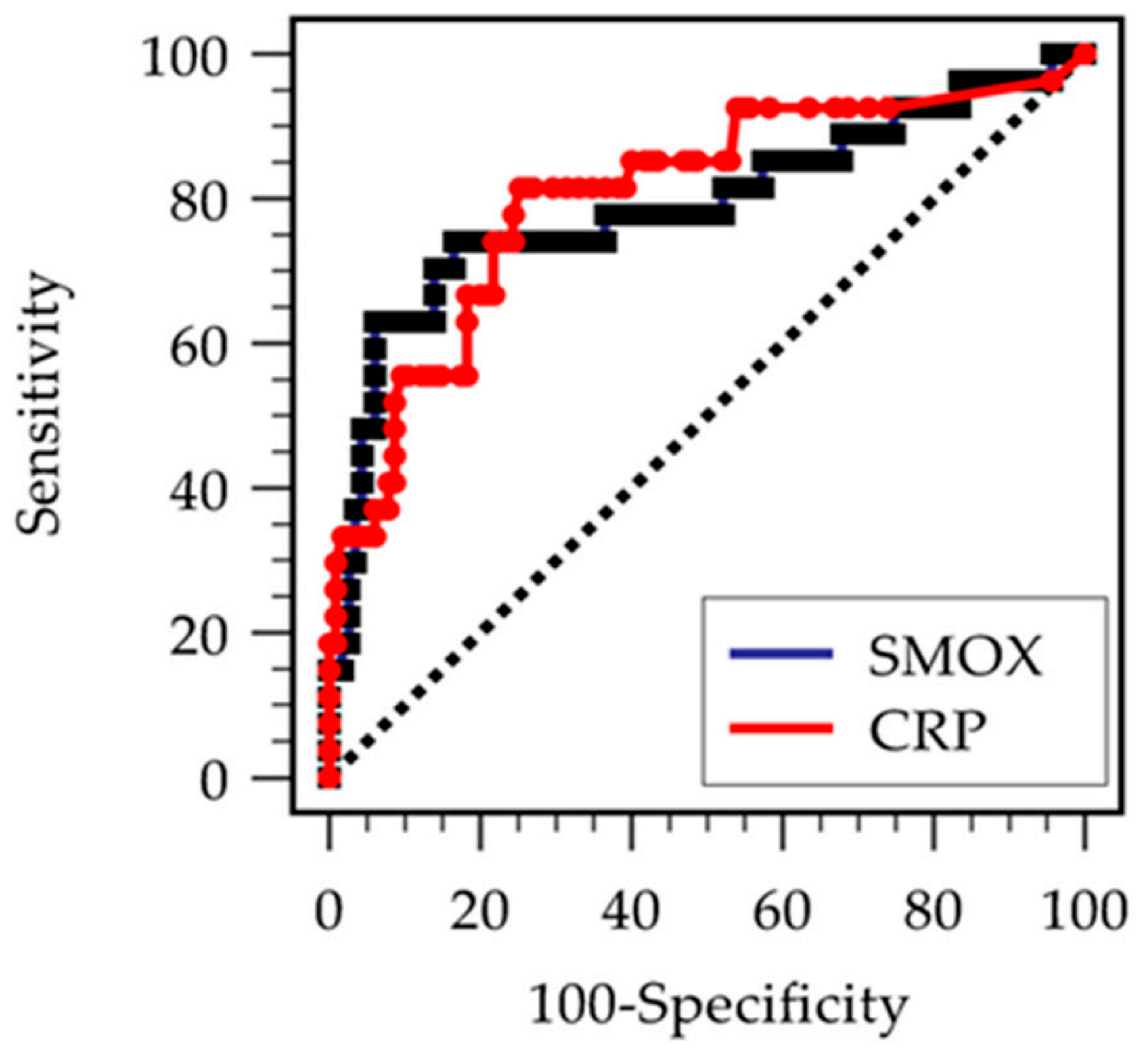
Receiver operating characteristic curves showing the discriminatory ability of serum SMOX and CRP for identifying PAD. The dashed diagonal line represents the line of no discrimination, corresponding to an AUC of 0.50.

The SMOX and CRP models revealed moderate-to-good discrimination for ABI-defined PAD, with AUCs of 0.790 (95% CI, 0.673–0.895; *p* < 0.001) and 0.804 (95% CI, 0.697–0.897; *p* < 0.001), respectively (Table 5). The combined SMOX–CRP model had the numerically highest AUC of 0.874 (95% CI, 0.785–0.946; *p* < 0.001), with a sensitivity of 0.889 and a specificity of 0.791 at a predicted probability cutoff of 0.132. At the exploratory cutoff values derived from the present cohort. For SMOX, an optimal cutoff of 28.40 ng/mL showed a sensitivity of 74.1% (95% CI, 53.7–88.9), specificity of 83.5% (95% CI, 75.4–89.7), positive predictive value of 51.3% (95% CI, 34.8–67.6), and negative predictive value of 93.2% (95% CI, 86.5–97.2). For CRP, an optimal cutoff of 0.41 mg/dL showed a sensitivity of 81.5% (95% CI, 61.9–93.7), specificity of 74.8% (95% CI, 65.8–82.4), positive predictive value of 43.1% (95% CI, 29.3–57.8), and negative predictive value of 94.5% (95% CI, 87.6–98.2). The combined SMOX–CRP model showed a sensitivity of 88.9% (95% CI, 70.8–97.6), specificity of 79.1% (95% CI, 70.6–86.1), positive predictive value of 50.0% (95% CI, 35.2–64.8), and negative predictive value of 96.8% (95% CI, 91.0–99.3). Pairwise AUC comparison showed that the combined SMOX–CRP model performed significantly better than SMOX alone (*p* = 0.036), but not significantly better than CRP alone (*p* = 0.154). These cutoff values and predictive indices were derived and evaluated in the same cohort and should therefore be interpreted as exploratory and cohort-specific rather than clinically established thresholds. Calibration was acceptable (Brier score, 0.100; Hosmer–Lemeshow, *p* = 0.522). After internal validation, the optimism-corrected AUC was 0.867, the optimism-corrected Brier score was 0.106, and the calibration intercept and slope were −0.052 and 0.952, respectively (Table 5). Figure 1 shows the receiver operating characteristic analysis of SMOX and CRP.

## 4. Discussion

In the present cross-sectional study of 142 patients undergoing maintenance HD, including 27 patients with ABI-defined PAD, we found that higher serum SMOX and CRP levels were associated with a low ABI. In the parsimonious multivariable model, prespecified to reduce the risk of overfitting, both SMOX and CRP remained independently associated with ABI-defined PAD after adjustment for age, whereas age did not. The directions and magnitudes of these associations were consistent across conventional logistic regression and the LASSO, ridge, and elastic-net sensitivity analyses. These findings suggest that the relationships of SMOX and CRP with a low ABI were not entirely dependent on a single model. Additionally, serum SMOX was inversely correlated with bilateral ABI and positively correlated with CRP, as determined by Spearman analysis. Nevertheless, given that only 27 patients had ABI-defined PAD, all multivariable estimates remain imprecise and should be considered exploratory.

The association between SMOX and ABI-defined PAD is biologically plausible in the context of the current study cohort. Patients undergoing HD are exposed to persistent oxidative stress secondary to uremia, chronic inflammation, dialysis-related bio-incompatibility, and impaired antioxidant defenses [22,23], which lead to endothelial dysfunction, aggravating the development of atherosclerosis and inducing vascular calcification [8]. SMOX catalyzes the oxidative conversion of spermine to spermidine, generating hydrogen peroxide and reactive aldehyde products, and excessive SMOX activity can promote oxidative injury, endothelial dysfunction, inflammation, and vascular remodeling [24]. In this setting, elevated circulating SMOX may be associated with a metabolic milieu accompanying vascular injury [25,26]. The inverse correlations observed between SMOX and both left- and right-side ABI support the relationship between higher SMOX levels and greater lower-extremity arterial impairment. However, circulating SMOX levels were measured only once, and whether SMOX directly contributes to vascular damage, represents a consequence of vascular injury, or primarily serves as a marker of systemic oxidative stress cannot be resolved from the present study findings.

In the present study, higher CRP levels were independently associated with ABI-defined PAD after multivariable adjustment, and an inverse association between ABI and CRP was observed. CRP is an important indicator of systemic inflammation [12]. Moreover, elevated CRP levels suggest an increased cardiovascular risk and can impact PAD progression in individuals with CKD [13], and uremic toxins and oxidative stress can amplify the inflammatory environment in these patients. Moreover, the positive relationship between SMOX and CRP observed in the present study suggests that oxidative stress and inflammatory signals may co-occur in patients with lower ABI. However, this finding should not be interpreted as evidence that SMOX and CRP act through a shared causal pathway. In patients with CKD, inflammation and oxidative stress are biologically interconnected processes that may contribute to endothelial dysfunction and cardiovascular complications, including peripheral vascular disease [8,27]. Finally, the AUC for both SMOX and CRP suggested moderate-to-good discriminatory performance for ABI-defined PAD in this cohort.

Previous studies have evaluated circulating biomarkers for ABI-defined PAD or low ABI in patients undergoing HD using ROC analyses. Zhou et al. reported that plasma pentraxin 3 (PTX3) showed an AUC of 0.901 for detecting ABI-defined PAD in HD patients, whereas hsCRP showed an AUC of 0.640 [28]. In another HD cohort, serum galectin-3 showed an AUC of 0.771, while CRP showed an AUC of 0.786, for identifying low ABI [10]. In the present study, the AUCs for SMOX and CRP were 0.790 and 0.804, respectively, and the combined SMOX plus CRP model yielded an AUC of 0.874, with a bootstrap-corrected AUC of 0.867. These findings suggest that SMOX may provide discriminatory information comparable to previously reported PAD-related biomarkers in HD and may offer additional information when combined with CRP. However, because these comparisons are indirect and based on different study populations, PAD definitions, and sample sizes, the incremental value of SMOX requires validation in external cohorts. Additional experimental and longitudinal studies are warranted to determine whether SMOX contributes to inflammatory signaling, whether inflammation increases SMOX activity, whether both reflect a common underlying uremic vascular process, or whether they are simply parallel markers of higher vascular risk.

Among conventional clinical factors, age may still have acted as a potential confounder in the present study. Patients with a low ABI were older than those with a normal ABI in the univariate analysis. In the correlation analysis, age was inversely correlated with both left- and right-side ABI and positively correlated with serum SMOX. Although age did not reach conventional statistical significance in the adjusted models, these findings support retaining age as an adjustment variable when evaluating the associations of SMOX and CRP with ABI-defined PAD. In the present study, we also found an inverse association between glucose and ABI. Patients with a low ABI had significantly higher glucose levels than those with a normal ABI, and glucose levels were inversely correlated with both right- and left-side ABI. Additionally, the prevalence of DM was higher among patients with a low ABI. These findings are supported by previous studies identifying DM as a major risk factor for PAD [29,30]. As a common underlying mechanism, long-term exposure to hyperglycemia results in increased levels of advanced glycation end-products and oxidative stress, leading to endothelial injury and accelerated atherosclerosis [31,32], which closely aligns with the global picture emerging from the present study, wherein conventional cardiovascular risk factors exert their influence together with inflammation and oxidative stress, leading to the deterioration of vascular health and the increased risk of PAD. However, glucose was no longer independently associated with PAD after multivariable adjustment; therefore, its role should be interpreted with caution. This association may be at least partially attributable to confounding by other relevant coexisting factors, and longitudinal studies are warranted to determine if glycemia directly contributes to PAD progression of over time in patients on HD.

Regarding nutritional status and dialysis adequacy, two issues commonly observed in patients undergoing HD, univariate between-group analysis showed that patients with a low ABI had lower serum albumin and creatinine levels as well as lower Kt/V and URR than those with a normal ABI. In the correlation analysis, bilateral ABI was positively correlated with serum creatinine and albumin, whereas the correlations of ABI with Kt/V and URR were small and not statistically significant. Therefore, albumin and creatinine may better reflect the nutritional-status component associated with ABI in this cohort, whereas the lower Kt/V and URR observed in the low ABI group should be interpreted as between-group differences rather than continuous correlation findings. Similarly to glucose, these parameters did not retain independent statistical significance in the multivariable analysis for ABI-defined PAD, likely due to their intercorrelations with other risk factors and inflammatory markers. These results indicate that, in patients on HD, ABI-defined PAD may be part of a broader high-risk clinical phenotype defined by inflammatory and oxidative stress, as well as less favorable nutritional and dialysis parameters [33,34]. Nonetheless, these associations should be interpreted with caution, given that this observational study was not designed to determine whether these markers were involved in PAD pathogenesis and merely reflect an overall illness burden in this population.

This study has several strengths. First, the study included a clinically important cohort of patients undergoing HD, utilized bilateral ABI measurements, and evaluated the consistency of the primary associations using regression, penalized regression, correlation, discrimination, and calibration analyses. Additionally, bootstrap internal validation was used to quantify model optimism. The associations of higher serum SMOX and CRP levels with increased odds of ABI-defined PAD were in a similar direction across the conventional and penalized regression models. The ridge and elastic-net regression shrink regression coefficients toward 0 and can reduce instability arising from a limited number of outcome events. Additionally, LASSO permits variable selection. The persistence of the associations observed for SMOX and CRP after coefficient shrinkage provides evidence of model stability. However, the penalized-model confidence intervals and *p*-values were derived using bootstrap procedures and should be interpreted as exploratory. Furthermore, the small number of ABI-defined PAD events, use of the same cohort for model development and internal validation, and uncertainty in selecting penalty parameters mean that these analyses do not eliminate the possibility of residual overfitting.

Nevertheless, the study limitations should also be acknowledged. First, the cross-sectional design precluded the determination of temporal or causal relationships. Second, the study was conducted in a single center using purposive sampling, limiting generalizability. Third, only 27 participants had ABI-defined PAD, resulting in limited statistical power and imprecise multivariable and calibration estimates. Fourth, serum SMOX and CRP were measured at a single time point and within-person variability could not be evaluated. Although laboratory personnel were blinded to the participants’ ABI status during SMOX and CRP measurement, and independent analytical validation of assay performance, including the limit of detection, dilution linearity, recovery, and comparison with a reference analytical method, was not performed. Therefore, some degree of measurement variability cannot be excluded. Fifth, the large number of exploratory correlation tests increased the possibility of chance findings. Sixth, PAD was defined using ABI without confirmation by vascular imaging. In patients undergoing HD, medial arterial calcification and reduced arterial compressibility may produce falsely elevated ABI values [35]. Although four patients with an ABI > 1.3 were excluded, this approach does not eliminate the possibility that patients with coexisting arterial occlusive disease and calcification had apparently normal ABI values and were classified into the non-low ABI group. Thus, an ABI within the study’s non-low range of 0.9–1.3 should not be interpreted as excluding underlying PAD. The low-ABI group may consequently represent a selected subset of patients with PAD detectable by ABI, limiting the generalizability of the observed associations to the broader HD population. These factors may have introduced disease misclassification and selection-related bias, the direction and magnitude of which could not be determined. Accordingly, our findings should be interpreted as associations with low ABI or ABI-defined PAD rather than with the entire spectrum of PAD. Additional pulse-wave recordings and toe–brachial index measurements were unavailable, precluding a sensitivity analysis using these measures to further assess potential misclassification. Although patients with an active infection at the time of blood sampling were excluded, information regarding previous or asymptomatic SARS-CoV-2 infection and COVID-19 vaccination status was not systematically collected. Therefore, residual effects of previous SARS-CoV-2 infection or vaccination on inflammatory and oxidative stress-related biomarkers, including CRP and SMOX, cannot be excluded. Finally, the internally derived SMOX and CRP cutoff values, biomarker thresholds and prediction estimates require external validation before clinical application and should not be considered clinically established thresholds at this stage. These limitations suggest that the current findings should be considered hypothesis-generating and require validation in larger prospective studies.

## 5. Conclusions

Higher serum SMOX and CRP levels were associated with ABI-defined PAD among patients undergoing maintenance HD. The observed associations among SMOX, CRP, glucose, and ABI suggest potential interrelationships among oxidative stress, inflammation, metabolic abnormalities, and lower-extremity vascular impairment. Although validation in larger prospective cohorts is necessary, these findings suggest that serum SMOX may be associated with ABI-defined PAD and could be further evaluated as a candidate biomarker in patients undergoing HD.

## Figures and Tables

**Table 1:** Clinical variables of the 142 hemodialysis patients with a normal ankle brachial index (ABI ≥ 0.9) or a low ankle brachial index group (ABI < 0.9).

Parameters	All Patients (*n* = 142)	Normal ABI Group (*n* = 115)	Low ABI Group (*n* = 27)	*p* Value
Clinical characteristics				
Age (years)	58.92 ± 12.30	57.43 ± 11.80	65.30 ± 12.57	0.002 *
HD duration (months)	69.36 (34.53–119.58)	70.92 (33.72–118.32)	64.20 (34.80–120.24)	0.897
Female, *n* (%)	65 (45.8)	49 (42.6)	16 (59.3)	0.118
Smoking, *n* (%)	17 (12.0)	12 (10.4)	5 (18.5)	0.244
Pre-HD body weight (kg)	68.88 ± 16.83	69.86 ± 17.59	64.71 ± 12.57	0.154
Body mass index (kg/m^2^)	24.96 (21.95–29.47)	25.23 (21.86–29.69)	24.47 (21.99–28.17)	0.845
Dialysis adequacy				
Kt/V (Gotch)	1.23 (1.20–1.36)	1.25 (1.20–1.39)	1.20 (1.19–1.25)	<0.001 *
Urea reduction ratio	0.71 (0.70–0.74)	0.72 (0.70–0.75)	0.70 (0.69–0.71)	<0.001 *
Hemodynamic parameters				
SBP (mmHg)	148.35 ± 22.44	147.24 ± 22.88	153.07 ± 20.18	0.226
DBP (mmHg)	79.60 ± 13.49	80.84 ± 13.68	78.74 ± 8.24	0.445
Left-ankle-brachial index	1.13 (1.01–1.18)	1.15 (1.07–1.22)	0.80 (0.71–0.86)	<0.001 *
Right-ankle-brachial index	1.12 (1.01–1.19)	1.14 (1.08–1.20)	0.80 (0.67–0.89)	<0.001 *
Laboratory parameters				
Hemoglobin (g/dL)	10.63 ± 1.19	10.66 ± 1.24	10.51 ± 0.97	0.566
Total cholesterol (mg/dL)	147.93 ± 41.91	148.09 ± 41.58	147.26 ± 44.09	0.927
Triglyceride (mg/dL)	136.00 (91.00–202.50)	125.00 (89.00–193.00)	142.00 (119.00–221.00)	0.130
Glucose (mg/dL)	145.00 (113.50–202.25)	139.00 (109.00–185.00)	197.00 (123.00–249.00)	0.005 *
Blood urea nitrogen (mg/dL)	63.94 ± 17.29	64.70 ± 17.11	60.70 ± 18.03	0.282
Creatinine (mg/dL)	10.08 ± 2.49	10.35 ± 2.52	8.94 ± 2.05	0.008 *
Albumin (g/dL)	4.30 ± 0.54	4.34 ± 0.54	4.10 ± 0.48	0.031 *
Total calcium (mg/dL)	9.28 ± 0.87	9.32 ± 0.81	9.09 ± 1.10	0.199
Phosphorus (mg/dL)	4.81 ± 1.41	4.84 ± 1.37	4.67 ± 1.61	0.578
iPTH (pg/mL)	329.55 (174.75–569.73)	315.50 (161.00–644.79)	334.30 (207.00–487.90)	0.845
C-reactive protein (mg/dL)	0.28 (0.12–0.61)	0.22 (0.10–0.42)	0.77 (0.43–1.30)	<0.001 *
Spermine oxidase (ng/mL)	21.06 (15.02–28.96)	19.54 (14.30–25.14)	35.68 (21.92–45.48)	<0.001 *
Underlying disease				
Diabetes mellitus, *n* (%)	70 (49.3)	51 (44.3)	19 (70.4)	0.015 *
Hypertension, *n* (%)	85 (59.9)	66 (57.4)	19 (70.4)	0.216
Glomerulonephritis, *n* (%)	49 (34.5)	42 (36.5)	7 (25.9)	0.297
Medication				
ARB, *n* (%)	35 (24.6)	30 (26.1)	5 (18.5)	0.412
β-blocker, *n* (%)	40 (28.2)	33 (28.7)	7 (25.9)	0.773
Calcium channel blocker, *n* (%)	43 (30.3)	36 (31.3)	7 (25.9)	0.584
Statin, *n* (%)	29 (20.4)	23 (20.0)	6 (22.2)	0.797
Fibrate, *n* (%)	31 (21.8)	26 (22.6)	5 (18.5)	0.643

Values for continuous variables are shown as mean ± standard deviation after analysis by Student’s *t*-test; variables not normally distributed are shown as median and interquartile range after analysis by the Mann–Whitney U test; values are presented as number (%) and analyzed by the chi-square test. Abbreviations: HD, hemodialysis; SBP, Systolic blood pressure; DBP, Diastolic blood pressure; Kt/V, fractional clearance index for urea; iPTH, intact parathyroid hormone; ARB, angiotensin receptor blocker. * *p* < 0.05 was considered statistically significant.

**Table 2:** Firth-penalized and conventional multivariable logistic regression analyses of factors associated with peripheral arterial disease.

Factors	Firth-Penalized Logistic Regression aOR (95% CI)	*p* Value	Conventional Logistic Regression aOR (95% CI)	*p* Value
SMOX, per 1 ng/mL	1.063 (1.018–1.115)	0.001 *	1.067 (1.019–1.117)	0.006 *
CRP, per 0.1 mg/dL	1.181 (1.079–1.321)	<0.001 *	1.198 (1.079–1.330)	<0.001 *
Age, per 1-year increase	1.041 (0.995–1.094)	0.082	1.044 (0.994–1.096)	0.083

The outcome was peripheral arterial disease, defined as a left or right ABI < 0.90 (27 events among 142 participants). Both models included age, serum SMOX, and CRP. Firth-penalized multivariable logistic regression was the primary analysis. Profile penalized-likelihood 95% confidence intervals and penalized likelihood-ratio *p*-values are reported for the Firth model. Conventional multivariable logistic regression with the same covariates was performed as a sensitivity analysis, using Wald 95% confidence intervals and Wald *p*-values. Adjusted odds ratios are presented for the specified increments of each variable. Abbreviations: ABI, ankle–brachial index; aOR, adjusted odds ratio; CI, confidence interval; SMOX, spermine oxidase; CRP, C-reactive protein. * *p* < 0.05 was considered statistically significant.

**Table 3:** Penalized logistic regression analyses for low ankle–brachial index (ABI < 0.9).

Factors	LASSO		Ridge		Elastic Net	
	aOR (95% CI)	*p* Value	aOR (95% CI)	*p* Value	aOR (95% CI)	*p* Value
SMOX, per 1 ng/mL	1.059 (1.022–1.132)	0.031 *	1.049 (1.025–1.093)	0.004 *	1.053 (1.025–1.103)	0.007 *
CRP, per 0.1 mg/dL	1.184 (1.090–1.339)	<0.001 *	1.167 (1.097–1.259)	<0.001 *	1.175 (1.097–1.283)	<0.001 *
Age, per 1-year increase	1.037 (0.996–1.107)	0.203	1.036 (0.996–1.081)	0.091	1.037 (0.995–1.090)	0.118

The outcome was peripheral arterial disease, defined as a left or right ABI < 0.90 (27 events among 142 participants). All models included age, serum SMOX, and CRP. Predictors were standardized before penalized modeling; penalized coefficients were transformed back to their original measurement units. Penalty parameters were selected by five-fold stratified cross-validation, minimizing log loss (LASSO C = 1.000; ridge C = 0.316; elastic net C = 0.464, L1 ratio = 0.10). For penalized models, 95% CIs were percentile bootstrap intervals, and *p*-values were bootstrap-Wald estimates based on 5000 stratified bootstrap samples with the selected tuning parameters held fixed. These inferential statistics for penalized regression should be interpreted as exploratory. Abbreviations: ABI, ankle–brachial index; LASSO, least absolute shrinkage and selection operator; aOR, adjusted odds ratio; CI, confidence interval; CRP, C-reactive protein; SMOX, spermine oxidase. * *p* < 0.05 was considered statistically significant.

**Table 4:** Spearman correlation coefficients between left ABI, right ABI, spermine oxidase, and clinical variables in 142 hemodialysis patients.

Variables	Left ABI		Right ABI		SMOX (ng/mL)	
	Spearman’s Coefficient of Correlation	*p* Value	Spearman’s Coefficient of Correlation	*p* Value	Spearman’s Coefficient of Correlation	*p* Value
Clinical and dialysis-related characteristics						
Age (years)	−0.276	<0.001 *	−0.287	<0.001 *	0.181	0.031 *
BMI (kg/m^2^)	0.080	0.345	0.090	0.285	−0.012	0.886
HD duration (months)	−0.012	0.889	−0.070	0.404	−0.051	0.547
ABI and SMOX measures						
Left-ABI	—	—	0.822	<0.001 *	−0.516	<0.001 *
Right-ABI	0.822	<0.001 *	—	—	−0.484	<0.001 *
SMOX (ng/mL)	−0.516	<0.001 *	−0.484	<0.001 *	—	—
Hemodynamic parameters						
SBP (mmHg)	−0.050	0.554	−0.004	0.961	−0.008	0.928
DBP (mmHg)	0.143	0.090	0.191	0.023 *	−0.202	0.016 *
Laboratory parameters						
Hemoglobin (g/dL)	−0.032	0.705	−0.056	0.509	0.029	0.736
Total cholesterol (mg/dL)	−0.100	0.238	0.050	0.555	0.110	0.192
Triglyceride (mg/dL)	−0.138	0.101	−0.145	0.086	0.120	0.155
Glucose (mg/dL)	−0.245	0.003 *	−0.228	0.006 *	−0.051	0.545
Blood urea nitrogen (mg/dL)	0.020	0.813	−0.010	0.906	−0.087	0.305
Creatinine (mg/dL)	0.318	<0.001 *	0.230	0.006 *	−0.255	0.002 *
Albumin (g/dL)	0.169	0.045 *	0.167	0.047 *	−0.169	0.044 *
Total calcium (mg/dL)	0.116	0.168	0.154	0.068	−0.096	0.255
Phosphorus (mg/dL)	0.012	0.890	−0.016	0.850	−0.124	0.141
iPTH (pg/mL)	−0.086	0.309	−0.094	0.263	0.067	0.427
CRP (mg/dL)	−0.305	<0.001 *	−0.314	<0.001 *	0.308	<0.001 *
Dialysis adequacy						
Kt/V (Gotch)	0.035	0.683	0.045	0.593	−0.135	0.109
URR	0.110	0.193	0.103	0.221	−0.118	0.161

Data are presented as Spearman’s rank correlation coefficient and two-sided *p*-value. * *p* < 0.05. Abbreviations: ABI, ankle-brachial index; BMI, body mass index; HD, hemodialysis; SMOX, spermine oxidase; SBP, systolic blood pressure; DBP, diastolic blood pressure; iPTH, intact parathyroid hormone; CRP, C-reactive protein; Kt/V, fractional clearance index for urea; URR, urea reduction ratio. * *p* < 0.05 was considered statistically significant.

**Table 5:** Discrimination and calibration of models for peripheral arterial disease in 142 hemodialysis patients.

Outcome	Model	Events/Total	AUC (95% CI) *p* Value	Sen (95% CI)	Spe (95% CI)	Brier Score	Hosmer– Lemeshow *p* Value	Bootstrap- Corrected AUC	Bootstrap- Corrected Brier Score	Bootstrap-Corrected Calibration Intercept	Bootstrap-Corrected Calibration Slope
PAD	SMOX	27/142	0.790 (0.673, 0.895) *p* < 0.001 *	0.741 (0.537, 0.889)	0.835 (0.754, 0.897)	0.115	0.624	0.790	0.118	0.034	1.018
	CRP		0.804 (0.697, 0.897) *p* < 0.001 *	0.815 (0.619, 0.937)	0.748 (0.658, 0.824)	0.116	0.356	0.804	0.119	0.006	1.004
	SMOX + CRP		0.874 (0.785, 0.946) *p* < 0.001 *	0.889 (0.708, 0.976)	0.791 (0.706, 0.861)	0.100	0.522	0.867	0.106	−0.052	0.952

Model discrimination for peripheral arterial disease was assessed using the AUC for serum SMOX, CRP, and a combined SMOX–CRP logistic regression model. The 95% confidence interval for the AUC was estimated using 5000 stratified bootstrap resamples. Calibration was assessed using the Brier score, Hosmer–Lemeshow goodness-of-fit test, calibration intercept, calibration slope, and a calibration plot. To reduce optimism bias and internally validate performance, 5000 stratified bootstrap resamples were used to estimate optimism-corrected AUC, Brier score, calibration intercept, and calibration slope. Peripheral arterial disease was defined as a left or right ankle–brachial index < 0.90. The *p*-value shown with the AUC tests discrimination against an AUC of 0.50. AUC, area under the receiver operating characteristic curve; Sen, sensitivity; Sep, specificity; CI, confidence interval; CRP, C-reactive protein; SMOX, spermine oxidase. A two-sided *p*-value < 0.05 was considered statistically significant; * indicates *p* < 0.05.

## Data Availability

The datasets generated and/or analyzed in the present study are available from the corresponding author upon reasonable request.

## Supplementary Materials

The following supporting information can be downloaded at: https://www.mdpi.com/article/10.3390/life16091521/s1, Table S1: Reduced Firth-penalized sensitivity analyses of factors associated with ankle–brachial index-defined peripheral arterial disease.

## Abbreviations

The following abbreviations are used in this manuscript:

ABI	Ankle-brachial index
aOR	Adjusted odds ratio
AUC	Area under the curve
BP	Blood pressure
BUN	Blood urea nitrogen
CI	Confidence interval
CKD	Chronic kidney disease
CRP	C-reactive protein
CVD	Cardiovascular disease
DM	Diabetes mellitus
HD	Hemodialysis
iPTH	Intact parathyroid hormone
Kt/V	Fractional clearance index for urea
LASSO	Least absolute shrinkage and selection operator
PAD	Peripheral arterial disease
SMOX	Spermine oxidase
TCH	Total cholesterol
URR	Urea reduction ratio
VIF	Variance inflation factors
