# Supplementary material for: Low Ankle-Brachial Index in Patients on Hemodialysis Is Associated with Elevated Serum Levels of Spermine Oxidase and C-Reactive Protein
Source: Life (Basel). 2026 Sep 13;16(9):1521. doi: 10.3390/life16091521 (PMC13608654; doi:10.3390/life16091521)
Supplement: Supplementary file 1 [file life-16-01521-s001.zip › life-4503619-supplementary.pdf]

**Supplementary Table S1.** Reduced Firth-penalized sensitivity analyses of factors associated with ankle–brachial index-defined peripheral arterial disease.

| Variable | Primary model | + Diabetes mellitus | + Albumin | + Kt/V |
|---|---|---|---|---|
| | aOR (95% CI)<br>*p* Value | aOR (95% CI)<br>*p* Value | aOR (95% CI)<br>*p* Value | aOR (95% CI)<br>*p* Value |
| SMOX, per 1 ng/mL | 1.063 (1.018–1.115)<br>$p = 0.001$* | 1.071 (1.024–1.126)<br>$p < 0.001$* | 1.063 (1.018–1.115)<br>$p = 0.001$* | 1.068 (1.016–1.130)<br>$p = 0.008$* |
| CRP, per 0.1 mg/dL | 1.181 (1.079–1.321)<br>$p < 0.001$* | 1.195 (1.090–1.334)<br>$p < 0.001$* | 1.178 (1.075–1.314)<br>$p < 0.001$* | 1.199 (1.084–1.379)<br>$p < 0.001$* |
| Age, per 1-year increase | 1.041 (0.995–1.094)<br>$p = 0.082$ | 1.045 (0.998–1.098)<br>$p = 0.061$ | 1.038 (0.991–1.091)<br>$p = 0.115$ | 1.076 (1.019–1.146)<br>$p = 0.007$* |
| Diabetes mellitus, present | - | 4.997 (1.575–19.625)<br>$p = 0.005$* | - | - |
| Albumin, per 1 g/dL increase | - | - | 0.569 (0.203–1.489)<br>$p = 0.254$ | - |
| Kt/V (Gotch), per 0.1-unit increase | - | - | - | 0.204 (0.062–0.505)<br>$p < 0.001$* |

The outcome was ABI-defined PAD, defined as a left or right ABI <0.90 (27 events among 142 participants). Firth-penalized multivariable logistic regression was used. The primary model included age, serum SMOX, and CRP. Diabetes mellitus, albumin, and Kt/V were added separately, one covariate at a time. Adjusted odds ratios are presented for the specified increments. Profile penalized-likelihood 95% confidence intervals and penalized likelihood-ratio *p* values are reported. URR was not included because of its strong mathematical and physiological overlap with Kt/V. All predictor VIFs in the reduced models were ≤1.193. *$p < 0.05$. Abbreviations: ABI, ankle-brachial index; aOR, adjusted odds ratio; CI, confidence interval; CRP, C-reactive protein; PAD, peripheral arterial disease; SMOX, spermine oxidase; URR, urea reduction ratio; VIF, variance inflation factor.
